# Progress in Medicine

**Published:** 1903-11-07

**Authors:** 


### Progress in Medicine.

### DISEASES OF CHILDREN.

**Infant Feeding.**—It is impossible in these columns even to enumerate the articles on this subject with which the medical press has been flooded, but we have pleasure in directing attention to a paper by Dr. Winters of New York.[1] Many are the pearls of scientific feeding which are found by enthusiastic explorers, but few stand the test of experience. Dr. Winters, like all the best authorities, is a strong supporter of breast milk as the proper diet, and on taking measures to secure this supply if it has been found to fail on a previous occasion. "With proper regulation of diet before confinement, and during the lying-in period, every mother can nurse successfully during the weeks when it is of so much moment to herself and her child that lactation should be stimulated." "Analysis and synthesis cannot provide a substitute having the composition and properties of mother's milk, at the beginning of lactation; a food adapted to the undeveloped and functionless state of the digestive organs, the immediate nutritive and developmental needs of a child at the threshold of life." He has no belief in machine-made cream, in which the fat globules are too large for the digestive powers of an infant, nor in milk which is made up in the laboratory on the percentage system. His own method is a home modification of milk, based on a dilution of the cream formed on standing. In the early weeks the upper half ounce of cream only is to be used, with the addition of milk sugar, lime water, and filtered water, and later on some of the subjacent milk is also used. Full tables are given of the exact proportions at the different ages. Pasteurization of milk is condemned because it dissolves the loose chemical union of mineral and proteid in milk, and as the result no formation of tissue for fresh growth can occur. "What God hath put together let not the pseudo-scientist cut wide asunder!" He claims that under his dietetic system, diarrhœa and cholera infantum never arise, and that sufferers from them can be better treated by this modified milk than by any other method. While some of the statements may be regarded as over-enthusiastic, we commend the study of his system to those who are not yet satisfied that the problems of infant feeding are solved.

**Congenital Cerebellar Ataxia.**—This is a somewhat rare affection which has probably in the past been classed amongst other forms of disease, but which has now been differentiated. It is defined by Dr. Batten[2] as "a disease of congenital origin starting in earliest life and characterised by marked unsteadiness of the head, trunk, and limbs, alteration in articulation, unsteadiness in sitting, in standing, and in walking, and by difficulty and slowness in swallowing." The general tendency of the disease is toward recovery. He relates eight cases, which are not all clearly of this type, but serve to illustrate how the symptoms of cerebellar ataxia may stand alone, or may be combined with those of a cerebral diplegia, and in some the cerebellar, in others the cerebral, symptoms may predominate. The diagnosis from hereditary ataxia (Friedreich's disease) is based on the facts that the latter is a family affection, usually develops about the tenth to the fifteenth year, and is accompanied by absent knee-jerks, spinal curvature, and pes cavus. The diagnosis from the hereditary cerebellar ataxia of Marie is easily made, as symptoms of the latter affection usually appear between the ages of 15 and 35 years. It may be easily confused with disseminated sclerosis, but this is an affection of extreme rarity in early life, and is always a progressive affection, whereas congenital cerebellar ataxy tends to be retrogressive.

**Infantile Scurvy.**—As a satisfactory explanation of the exact cause of this affection has not yet been forthcoming, the results of an investigation into this question pursued by Dr. Robert Hutchison[3] are interesting. He finds that if an infant has developed scurvy on a certain diet, and to this diet we simply add fresh fruit juice, the child will get well. This shows that it is not any special constituent, present in the food, which is injurious, but it is the absence of a special constituent which produces the disease. Some have held that a special ferment is absent from the food, but if the fruit juice is boiled, a proceeding which would destroy any ferment, it will act quite as well. He next tested the question as to whether the anti-scorbutic element was crystalline or colloid, and found that the dialysate had a curative effect while the colloid constituents had not. This suggested the use of citrate of lime and citrate of potash, but he did not find that they, even in large doses, had a curative effect. On the other hand he found that from a mixture containing malate of potash, citrate of potash, and tartrate of potash he obtained great improvement, which, however, was not brought about so quickly as by the use of fruit juices and fresh diet.

**Convulsions.**—Dr. John Thomson[4] discusses the causation, prognosis, and treatment of convulsions in young children. The immediate treatment, in a mild attack, consists of the employment of a hot bath, with or without mustard, or of a mustard pack. When the fit lasts longer, or is repeated at short intervals, the administration of chloroform is called for. Other methods that may be used are the rectal injection of chloral or the hypodermic injection of morphia. The question of causation is a very large one, and many possible local and general causes must be kept in mind and inquired into. He notes that an inherited nervous constitution may be present, as shown by the occurrence of fits in early life in successive members of the same family and their descendants. In some cases no organic or peripheral cause can be discovered, and these are described as "idiopathic convulsions." The fits commence sometimes during the first week of life, and generally within the first month or two. They occur with increasing frequency, and may amount to 30 or even 40 in a day. More radical treatment is called for here, and what he recommends is the use of chloral in frequently repeated doses until the child is under its influence. For a baby a week old, 1 grain may be given every two hours, and for older children 2 grains is not too large a dose. This is continued until the fits have entirely ceased for six or eight hours, and if not speedily successful the dose can be increased until the child begins to get too drowsy to swallow his milk. The chloral is then given in doses diminishing in amount and frequency. Provided there is no organic trouble behind these fits the prognosis is good, although the combined effect of the fits and the chloral may lead to a temporary dulling of the cerebral activity. Attacks of petit mal are more serious from a prognostic point of view than severe convulsions, for they are often a sign, and the only one, of serious cerebral defect. Dr. Eustace Smith[5] has found that growing boys and girls may suffer from reflex convulsions without there being any actual brain disease or epilepsy. Such children usually possess a highly-strung nervous system, partly due to inheritance, and may have suffered from convulsions in infancy. The important point in the treatment is to find out and relieve the peripheral source of irritation. He lays stress on the fact that this is frequently moderate in degree but of some duration, so that the system has been kept for some time in a state of unrest before the outbreak occurs. Constipation, with excessive discharge of mucus, eye-strain, and cold feet, are instances of the chronic irritation to which he refers.

**Intussusception.**—A case of chronic intussusception, successfully treated by operation, is reported by Mr. R. G. Hogarth.[6] The patient, a girl of six years, after an attack of diarrhœa was seized with acute abdominal pain, occurring in paroxysms. Later came vomiting, thirst, and loss of flesh, but at no time was there obstruction, or the passage of blood or slime. Six weeks after the onset she was admitted into hospital, where Mr. Hogarth found a swelling passing along the line of the transverse and descending colon, and proceeded to operate. He found and easily reduced a large intussusception, with the exception of the last part which proved to be the appendix invaginated into the cæcum. The intussuscepted appendix was partly reduced and then removed in the ordinary way. This is a striking case from the length of the illness, the absence of adhesions, the unusual starting place in the appendix, and the successful result. Mr. D'Arcy Power[7] records a case of ileo-iliac intussusception in an infant of six months which was spontaneously relieved. The symptoms were clearly those of intussusception, and when the abdomen was opened, a portion of the small intestine about three inches long, deeply congested, with marks of constriction at one part, was found. No other lesion was found, and the only conclusion he could draw was that reduction of an intussusception had taken place before operation. Very few similar cases are on record, although Mr. Power suggests that the sudden attacks of colic in infants may sometimes have a similar pathology.

**Melæna Neonatorum.**—Dr. E. Fuhrman,[8] having found all the existing methods of treatment useless in this very fatal affection, was led to try the subcutaneous injection of gelatine, with the result that two out of three cases recovered. The treatment should be adopted as soon as the bleeding is discovered. The following formula is recommended:—℞ Gelatinæ albæ, 1·0 gram.; sodii chloridi, 0·3 gram.; aquam dist. ad 50·0 gram. Of this 2 per cent. solution not less than 12 to 14 drachms should be injected. Writing on the use of subcutaneous injections of sterilized gelatine in the spontaneous hæmorrhages of new-born children, Dr. T. A. Abt[9] sounds a note of warning as to the sterilization of this substance. It has been found that toxic substances, probably ptomaines, are frequently present, but if applied locally on a compress, or given by the mouth, the gelatine is innocuous.

**Tuberculous Peritonitis.**—A discussion on this subject took place at a meeting of the Society for the Study of Disease in Children.[10] Several of the speakers held that the prognosis, in the absence of certain complications, was good, and that surgical interference was not called for. In reply to these statements Mr. Watson Cheyne said that the surgeons were usually called in by the physicians, who said there was no other method of cure. If the physicians could cure these cases by medical measures, he would accept their statements. He was not prepared to deny the value of laparotomy in this condition, although he admitted that he could give no explanation of the manner in which it acted.

**Adherent Pericardium.**—Dr. G. M. Swift[11] has seen 18 cases of this condition in hospital practice, all of whom died. Many cardiac murmurs may be present, without valvular disease, owing to the manner in which the heart is held open by the firm union to the chest wall. Enlargement of the liver was frequently the first sign of the disease, and the action of the heart usually became turbulent. The use of cardiac stimulants often had a bad effect. The most common cause was rheumatism. In the hospital cases death occurred on an average in about five years after the diagnosis of adherent pericardium had been made.

[1] Med. Rec., March 7, 1903, p. 366. [2] Clin. Jour., May 27, 1903, p. 81. [3] Clin. Jour., March 25, 1903, p. 365. [4] Internat. Clin. Vol. I. Twelfth Series. 1903. [5] Lancet, Jan. 24, 1903. [6] Brit. Med. Jour. Vol. I., 1903, p. 850. [7] Ibid., April 25, 1903, p. 964. [8] Ep. Brit. Med. Jour., March 7, 1903, and Münch. Med. Woch., Sept. 2, 1902. [9] Bost. Med. and Surg. Jour., Jan. 29, 1903. [10] Lancet, Jan. 17, 1903. [11] Med. Rec., Nov. 29, 1902, p. 876.

